# Chemical Communication in Artificial Cells: Basic Concepts, Design and Challenges

**DOI:** 10.3389/fmolb.2022.880525

**Published:** 2022-05-26

**Authors:** Hedi Karoui, Pankaj Singh Patwal, B. V. V. S. Pavan Kumar, Nicolas Martin

**Affiliations:** ^1^ Univ. Bordeaux, CNRS, Centre de Recherche Paul Pascal, UMR 5031, Pessac, France; ^2^ Department of Chemistry, Indian Institute of Technology Roorkee, Roorkee, India

**Keywords:** artificial cells, chemical signaling, bottom-up synthetic biology, collective behaviours, dynamic colloidal systems, systems chemistry

## Abstract

In the past decade, the focus of bottom-up synthetic biology has shifted from the design of complex artificial cell architectures to the design of interactions between artificial cells mediated by physical and chemical cues. Engineering communication between artificial cells is crucial for the realization of coordinated dynamic behaviours in artificial cell populations, which would have implications for biotechnology, advanced colloidal materials and regenerative medicine. In this review, we focus our discussion on molecular communication between artificial cells. We cover basic concepts such as the importance of compartmentalization, the metabolic machinery driving signaling across cell boundaries and the different modes of communication used. The various studies in artificial cell signaling have been classified based on the distance between sender and receiver cells, just like in biology into autocrine, juxtacrine, paracrine and endocrine signaling. Emerging tools available for the design of dynamic and adaptive signaling are highlighted and some recent advances of signaling-enabled collective behaviours, such as quorum sensing, travelling pulses and predator-prey behaviour, are also discussed.

## 1 Introduction

Living cells are compartmentalized chemical systems that continuously sense their environment and interact with other cells (Bradshaw and Dennis, 2010). Communication is essential for cells to survive by recognizing and cooperating with helper cells while avoiding and fighting against competitor cells. Communication is also crucial to coordinate individual cellular responses in cell populations or in multicellular organisms and to achieve higher-order collective behaviours.

Biologists commonly exploit signaling pathways to control cellular behaviours using well-established biomolecular tools, such as genome engineering (Hennig et al., 2015). Emerging top-down synthetic biology studies have also started rationally designing and rewiring communication networks within or between living cells (Yokobayashi et al., 2002; Dueber et al., 2003; Isaacs Farren et al., 2003), in particular to perform specific tasks, such as coordinated density-dependent responses (Haseltine Eric and Arnold Frances, 2008), biological computation (Benenson, 2012), regulated killing (You et al., 2004) or conversion of waste materials into valuable products (Alper and Stephanopoulos, 2009; Lu et al., 2019; Sanford and Woolston, 2022). However, the cellular complexity, poor understanding of metabolic pathways, biological processes and unknown sources of “noise” in gene expression make top-down synthetic biology often very challenging. Living cells also function under specific conditions (e.g., of pH, salinity or temperature) which may limit their utilization, and different cells may compete for resources or interfere with each other (cross-talk).

In that context, the design and construction of artificial cells offers avenues to the bottom-up engineering of precisely controlled communication pathways between synthetic chemical compartments. Artificial cells designate man-made functional micro-compartments inspired by living cells and assembled from a small number of well-defined building blocks (Ishikawa et al., 2004; Noireaux and Libchaber, 2004). Although first thought as strictly biomimetic compartments delimited by a phospholipid bilayer, artificial cells now encompass a palette of systems, including vesicles delineated by a polymer (Discher Bohdana et al., 1999; Marguet et al., 2013; Buddingh’ and van Hest, 2017; Rideau et al., 2018) or protein (Huang et al., 2013) shell (polymersomes and proteinosomes, respectively), water-transferred cross-linked Pickering emulsions (Li et al., 2013) (colloidosomes) and other types of capsules (Douliez et al., 2019) or membrane-free microdroplets produced by liquid-liquid phase separation (Koga et al., 2011; Martin, 2019) (such as complex coacervates). The terms “protocells”, “synthetic cells”, “artificial cells” or “minimal cells” have often been used to designate such bio-inspired micro-compartments, although with certain nuances, e.g., depending on whether the assemblies are built using purely synthetic parts, biological modules or prebiotically relevant molecules, or depending on the ultimate goal of the system (e.g., re-engineering biological functionalities, deciphering the origins of life, building new smart materials). In this review, we refer to all these systems as “artificial cells” regardless of their cytomimetic relevance and function.

Over the past few years, research has gradually moved from the design of single artificial cells towards the study of populations of artificial cells able to interact, cooperate, or compete via chemical signaling. Artificial cells provide a unique opportunity to strip-out the complexity of cell signaling, build robust, rationally designed communication pathways, and develop a quantitative theoretical framework of molecular communication by testing hypothesis in well-defined systems. Advances geared towards controlling synthetic cell communication have recently been reviewed (Smith et al., 2022). The construction of artificial cells that can communicate with each other could also lead to the emergence of a new class of smart colloidal materials capable of a diverse range of behaviours depending on their local environment, which can be defined by parameters such as presence of signaling compartments in the vicinity, diversity of signals and signaling objects, chemical gradients, or population density. Communicating artificial cells would also be interfaced with living materials to perform desired tasks, as reviewed elsewhere (Lentini et al., 2016; Mukwaya et al., 2021).

After introducing general concepts of cell signaling, we discuss in this review recent studies on communication between artificial cells through the prism of the mode of communication and signaling distance. Emerging directions geared at dynamically controlling artificial cell signaling are also presented, together with the achievement of collective behaviours and future challenges in the field.

## 2 Chemical Communication in Biology: Basic Concepts

**FIGURE 1:**
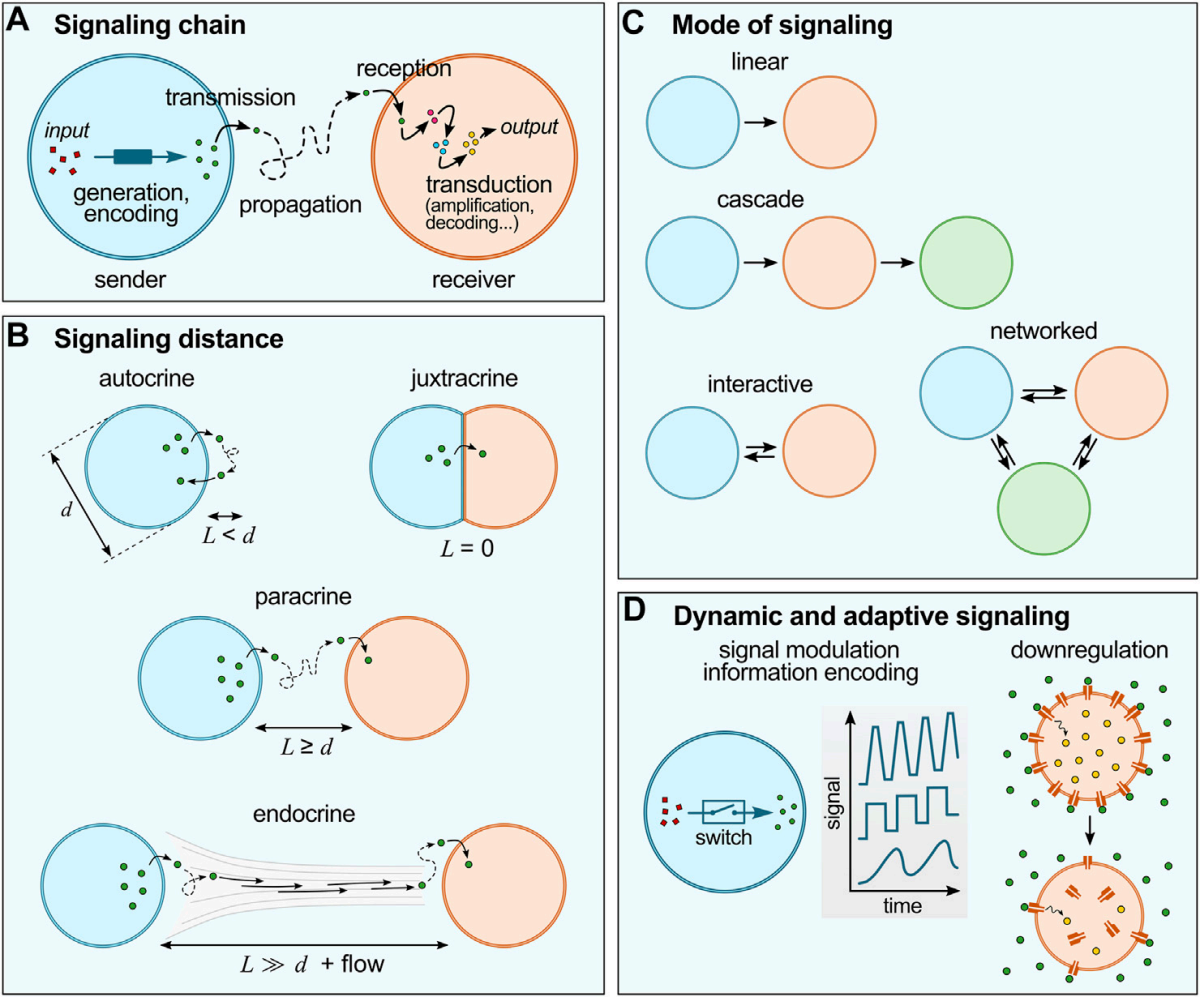
Basic concepts of cell chemical signaling. **(A)** The signaling chain include a sender and a receiver cell, a signal (generated and encoded in the sender cell; and transduced within the receiver cell) and a propagation medium. **(B)** Chemical signaling can be characterized by the distance between sender and receiver cells (*L*) compared to the typical size of the cells (*d*). **(C)** Different modes of signaling are possible, from simple linear or cascade modes to more sophisticated interactive of networked modes. **(D)** Cell signaling is dynamic and adaptive: for instance, living cells utilize switches to regulate signal production (concentration, frequency, etc.), and advanced reconfigurable systems to regulate signal responses, such as receptor internalization.

Communication in artificial cells builds upon well-established phenomenological knowledge of biological cell signaling, which is the capacity of a cell to receive, process and transmit signals (Bradshaw and Dennis, 2010). While signals can directly originate from the environment (i.e. without a sender cell), cell-to-cell communication involves four components: a sender and a receiver cell, a signal and a medium though which it propagates (Figure 1A). The sender cell generates, encodes and transmits a signal that propagates through a medium and triggers a response in a receiver cell via transduction signaling cascades used to decode and process the signal. A great variety of signals are sensed and used by cells, including electrical signals (Catterall et al., 2017), mechanical forces (del Rio et al., 2009; Coste et al., 2010) or heat (Caterina et al., 1997), but the most abundant form of cell communication is chemical signaling, whereby diffusible chemical messengers act as signals to exchange information. Cells are indeed highly sophisticated chemical systems that can readily synthesize, degrade and process molecules using biochemical or binding reactions.

Chemical signaling in living cells can be characterized by (i) the communication chain and mode of communication, (ii) the distance between sender and receiver (or signaling distance) and (iii) the adaptive nature of signaling networks:

(i) The communication chain includes signal generation, encoding, transmission, propagation, reception and decoding (Figure 1A) (Bradshaw and Dennis, 2010). In living cells, signal generation and encoding is performed by the synthesis and/or release of molecules to the outer environment by passive diffusion or active transport across the membrane. Propagation typically relies on Brownian motion, possibly coupled to flows of the external medium. Reception starts by the binding of the molecular signals to their receptors to trigger transduction cascades involving intracellular messengers so as to produce the desired response, such as selective gene activation. According to communication theories, the flow of information between different networked points follows a specific pathway described by the mode of communication (Figure 1B) (de Luis et al., 2021). The simplest one is the linear model, whereby information is transmitted unidirectionally from the sender to the receiver through a specific medium. Cascade models are concatenated linear pathways in series, where some compartments act both as senders and receivers to pass on information in one direction. Interactive models of communication also involve compartments that act both as senders and receivers, but that exchange information in a bidirectional way, so that a feedback is observed, from the receiver back to the sender. More intricate networks can be envisioned when consortia of cells are involved.
(ii) Cell signaling also depends on the distance between sender and receiver cells (Figure 1C) (Bradshaw and Dennis, 2010), and is described as autocrine when the signal produced by a cell affects the cell itself, juxtacrine when communication requires close contact between cells, paracrine when signals produced by a cell diffuse locally and only affect neighboring cells, and endocrine when signals propagate on longer distances via the circulatory system to affect distant receiver cells. Importantly, the signaling distance sets a characteristic time for chemical communication and may modulate the signal intensity. Since molecular diffusion from one cell to another typically follows normal diffusion (unless a directional flow is involved), the further cells are apart, the longer it takes for the signal to reach its target. For a fixed concentration of signaling molecules and fixed binding constant to their receptor in the receiver cell, the weaker the outcome of signaling may become with increasing distance (due to dilution).
(iii) Last, cell signaling is dynamic and adaptive (Bradshaw and Dennis, 2010) (Figure 1D). Living cells are highly evolved communicating compartments that dynamically alter signaling pathways depending on their environment and history. For instance, molecular switches are broadly used by cells to temporarily activate and inactivate reactions and thus to dynamically modulate the intensity, speed or frequency of signal generation or transduction (Ghusinga Khem et al., 2021). Another well-established example of dynamic signaling regulation is the process known as receptor internalization, whereby exposure of cells to an excessive amount of ligand can induce downregulation via trafficking of membrane receptors to the cytoplasm, (Katzmann et al., 2002) which decreases the cells sensitivity to the ligand molecule. Collective cellular behaviours also rely on adaptive strategies since they require careful sensing of the environmental conditions: for instance, the coordinated communication known as quorum sensing in bacteria (Mukherjee and Bassler, 2019) involves changes in the local bacterial population density via energy-fueled (active) processes (e.g., bacterial self-reproduction or migration).

Building upon these well-established characteristics of biological cell signaling, we discuss below examples of their realization in artificial cells.

## 3 Compartmentalization and Communication Chain in Artificial Cells

### 3.1 Artificial Cell Compartmentalization

Artificial cells are man-made, cell-sized compartmentalized chemical systems. Compartmentalization is central to localize chemical species and reactions and sustain chemical gradients (Buddingh’ and van Hest, 2017). In the context of chemical communication, artificial cell-like chassis can be classified based on their capacity to exchange molecules with their environment, which mainly depends on the nature of their boundary. Two classes of cell-like constructs can be distinguished based on the presence or absence of a membrane to delimit the compartments (Martin and Douliez, 2021). Membrane-bounded compartments include vesicles and capsules where a physical barrier delimits an aqueous lumen, and can be divided into two sub-classes, depending on whether the membrane is continuous (as in liposomes or polymersomes) or discontinuous (i.e., porous, as in colloidosomes, proteinosomes, etc.). The compartments with continuous membrane allow diffusion of species with specific physicochemical properties (e.g., small apolar species can diffuse through lipid bilayers (Mansy, 2010)), those having a discontinuous membrane typically limit diffusion of solutes based on the size of their pores (e.g., only small molecules can diffuse through a proteinosome shell) with a well-defined molecular weight cut-off (Huang et al., 2013). In comparison, membrane-free compartments are microdroplets produced by segregative or associative liquid-liquid phase separation (LLPS) in water (Martin, 2019). The seminal example of associative LLPS is complex coacervation, where the entropy-driven association of oppositely charged polyions produces highly hydrated, polymer-rich, liquid-like droplets suspended in a dilute continuous aqueous phase (Sing and Perry, 2020; Dinic et al., 2021; Kapelner et al., 2021). These membrane-free compartments have emerged as interesting compartments to build protocells (Koga et al., 2011; Ghosh et al., 2021), artificial cells (Crowe and Keating, 2018; Martin, 2019; Wang X. et al., 2021) and models of membraneless organelles (Yewdall et al., 2021). Importantly, they selectively sequester or exclude species depending on their charge, hydrophobicity, size, or binding motifs, but these solutes usually remain in dynamic equilibrium with their environment, which means they can diffuse in or out of the droplets with an average residence time that relates to their partition coefficient (Jia et al., 2014).

Signal transmission and reception will therefore differ depending on which compartment is used to assemble artificial cells. For instance, continuous membranes may require the insertion of channels (e.g. protein pores) to allow diffusion of molecules. In contrast, the need for insertion of pores can be eliminated if the membrane is porous enough (Booth et al., 2019). For instance, by forming proteinosomes made up of a cross-linked monolayer of bovine serum albumin and poly (N-isopropylacrylamide) nanoconjugates (Huang et al., 2013; Huang et al., 2014), small molecules such as glucose (Martin et al., 2018) or ATP (Booth et al., 2019) but also oligonucleotides (Joesaar et al., 2019) can diffuse freely across the membrane. In membrane-free compartments, selective sequestration and partition coefficients will determine the ability of signaling molecules to diffuse in and out of the droplets.

### 3.2 Enabling Reactions for Signal Production and Processing

Studies reporting chemical signaling in artificial cells have used different molecular messengers, including enzyme cascade intermediates (Buddingh’ et al., 2020), transcription regulators (Dupin and Simmel, 2019), proteins (Niederholtmeyer et al., 2018) and short DNA/RNA strands (Joesaar et al., 2019). Three main classes of enabling reactions have been used to produce or process these signaling molecules in artificial cells: enzyme-mediated catalysis (Buddingh’ et al., 2020), cell-free gene-directed protein expression (Niederholtmeyer et al., 2018), and DNA nanotechnology-based processes (Joesaar et al., 2019) (such as DNA strand displacement reactions). These biochemical or bio-inspired reactions offer great modularity, robustness, selectivity and high efficiency. Interestingly, they also rely on molecules with very different sizes, which may set specific signaling times since low molecular weight substrates diffuse faster than larger species such as proteins or DNA (typical diffusion coefficients may span 2-3 orders of magnitude; for instance D_H2O2_ ∼ 2 × 10^–5^ cm^2^ s^−1^ while D_dsDNA_ ∼ 5 × 10^–7^ to 0.81 × 10^–8^ cm^2^ s^−1^ for ∼20 bp) (Lukacs et al., 2000; Tjell and Almdal, 2018).

Importantly, compared to other types of signals that may require frequency or intensity modulation to encode a message, molecules may readily encode information in their nature or structure: their specificity will induce a specific response in the receiver compartment. For instance, small molecular substrates selectively activate their matching enzyme to produce a desired product, sequence-defined polynucleotide strands will selectively bind to their complementary partner, etc. The binding constant between signaling molecules and their target will also impact the efficiency of communication. Additional encoding of information can be obtained by modulating the concentration of molecules signals, the level or frequency of protein activation, etc., although this has not yet been thoroughly demonstrated in artificial cells.

## 4 Distance-Based Classification of Artificial Cell Communication

Distance plays an important role in limiting the sphere of influence of chemical signaling via dilution of signals in the absence of any amplification motifs. We here introduce two characteristic length-scales to classify signaling between artificial cells (Figure 1B): *L* as the distance between the sender and receiving cell, and *d* as the typical cell size. Accordingly, autocrine signaling occurs for L<d (the sender cell is also the receiver cell and influences its own behaviour), juxtracrine signaling corresponds to L=0 (sender and receiving cells are in direct contact with each other), paracrine signaling refers to L≥d (sender and receiver cells are in proximity without direct contact), and endocrine signaling occurs for L≫d (sender and receiver cells are distant and signaling molecules are aided by flows of the surrounding medium in addition to Brownian motion). These different length scales of signaling will have different time scales of response: considering only Brownian motion (no external flows), the characteristic time of communication scales with the square distance between sender and receiver cells: *τ* = *L* ^2^/*D*, where *D* is the diffusion coefficient of the signaling molecules. For instance, if we compare autocrine (*L* < *d*–let’s fix *L* = 0.2*d*) and paracrine (*L* ≥ *d*–let’s fix *L* = 2*d*) signaling for a given diffusible molecule (i.e., a fixed diffusion coefficient *D*), we get: τ_paracrine_ ∼ 100 × τ_autocrine_, namely, the characteristic timescale for paracrine signalling is ca. 100-fold longer than for autocrine signaling. Although this very simplistic consideration does not take into account permeation of signaling molecules through membranes, their concentration or binding constant to their target, it is consistent with theoretical modelling of autocrine and paracrine trajectories (Coppey et al., 2007; Berezhkovskii et al., 2008). We briefly highlight below examples of each class of signaling.

### 4.1 Autocrine Signaling

Autocrine signaling is a way of communication of a cell to itself or to other cells of the same type, that we can also call “self-communication” ( L<d ). It is ubiquitous in bacterial as well mammalian cells, where specific membrane receptors receive signals emitted from the cell itself, which helps them do advanced functions, such as embryo development, proliferation of T cells (Doğaner et al., 2016) or tumor formation (Grivennikov and Karin, 2008). Since artificial cells are minimal representations of real cells, the self-induced functional/structural changes are usually much simpler than in living cells. Yet, there are quite a few examples of “self-communication” in artificial cells, although they may not have been described as such in the literature. Very broadly, we have classified examples in literature where self-induced functional/structural changes were observed as “autocrine” signaling or “self-communication”.

**FIGURE 2:**
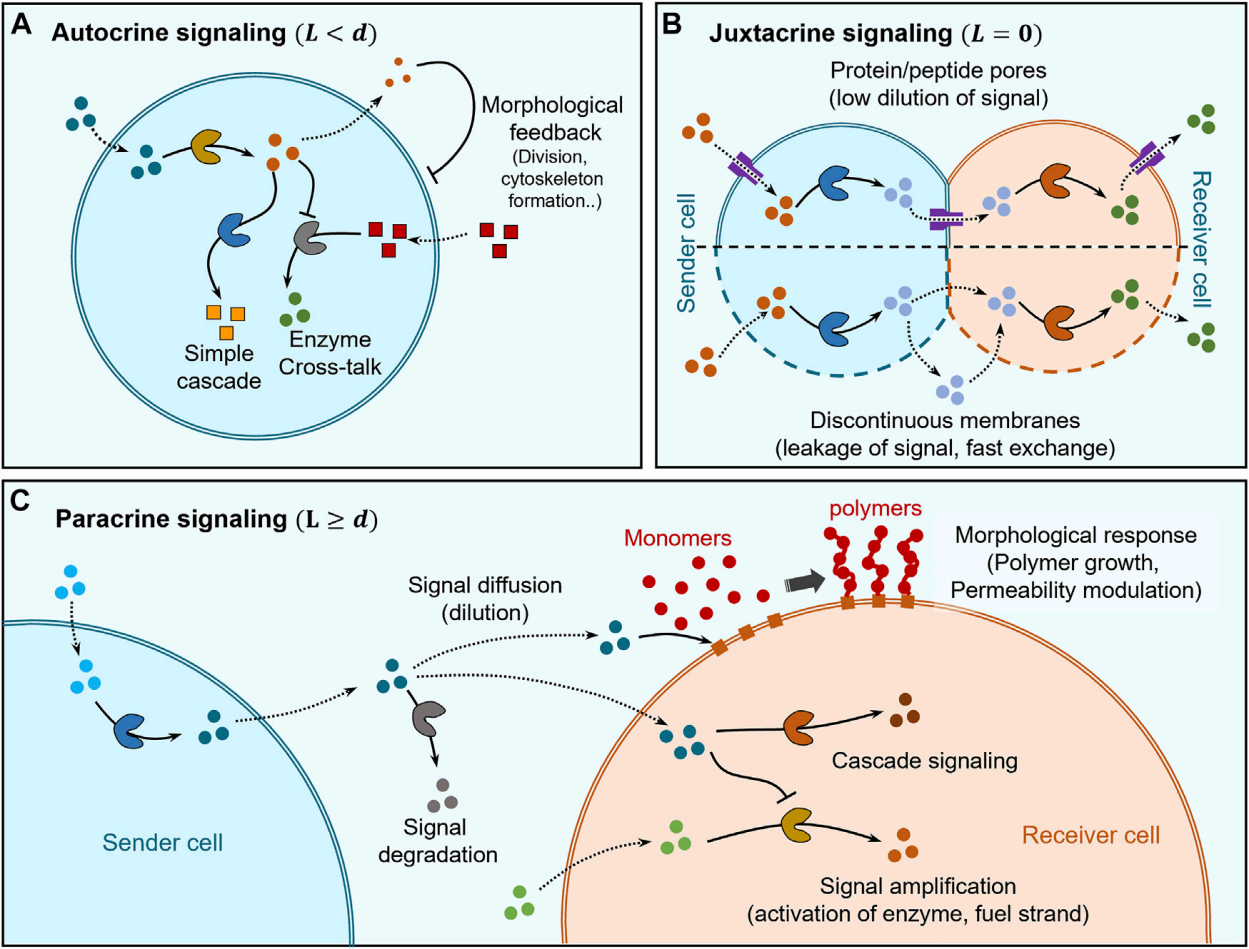
Distance based classification of signaling. **(A)** Self-signaling (autocrine signaling) within artificial cells can be mediated by simple enzyme cascades and enzyme cross-talk to self-modulate chemical behaviour while morphological changes can also be elicited via cytoskeleton formation and division into second-generation cells. **(B)** Juxtacrine signaling occurs between sender and receiver cells that are in direct contact and its regulation depends on the type of membranes separating them. In the case of sender and receiver cells having continuous membranes (lipid bilayers), the insertion of protein/peptide pores into the membrane allows regulation of communication between themselves and with the environment while also avoiding dilution of signaling molecules due to limited pore channels. Sender and receiver cells having discontinuous membranes exhibit fast-unchanneled exchange of signaling molecules between themselves and the environment. **(C)** Paracrine signaling occurs between sender and receiver cells which are separated by distances larger than their size. The signaling molecules get diluted and may be degraded before reaching the receiver cell, where they can trigger a chemical response via cascade signaling or undergo amplification for increasing the signaling distances. Signaling molecules can also trigger morphological responses, for e.g., via activating membrane-immobilized chain growth agents leading to polymer brush growth on the membrane of the receiver cell allowing modulation of permeability.

The first type of autocrine signaling corresponds to artificial cells hosting enzyme cascades, since the product of enzymatic transformation acts as a signaling molecule for the next enzyme in the cascade (Figure 2A). In a seminal example, Zhao et al. hosted a three-enzyme cascade within a proteinosome constituted by β-galactose (β-gal), glucose oxidase (GOx) and horseradish peroxidase (HRP) (Li et al., 2013). Co-localization of the enzymes within the same artificial cell enabled high reaction efficiency due to the short diffusion length between successive enzymes. Co-localization of enzymes has also been exploited to increase crosstalk between enzymes leading to modulation of their activity (Jiang et al., 2022). In an example, Appelhans et al. constructed polymersome-in-proteinosome multi-compartmentalized artificial cells that used GOx-catalysed proton secretion as a signal to control local pH conditions and modulate the activity of alkaline phosphatase (ALP; Figure 2A). This model mimicked homeostasis behaviour with the ability to sense glucose concentrations below normal blood glycemic condition (Wang D. et al., 2021). Recently, Sun and co-workers developed a dual enzyme-containing tourmaline microparticle colloidosome system that showed cascade cycling of signaling molecules (Lv et al., 2020). In this work, alcohol dehydrogenase (ADH) catalysed the reduction of NAD^+^ to NADH which then acted as a signaling molecule for the GOx-tourmaline microparticle system, which converted it back to NAD^+^. This reaction system mimicked the redox cycle of nicotinamide cofactor (NAD^+^/NAD) that frequently occurs in biological systems.

Autocrine signaling has also been used to trigger changes in shape, structure and even cause division of artificial cells (Figure 2A). Martin et al. demonstrated multi-compartmentalised artificial cells (proteinosome-in-coacervate) that could self-induce morphological transformations (Martin et al., 2018). In this system, GOx-mediated gluconic acid production was used to release protons as signal and the resultant self-induced local pH change was sensed by the coacervate to induce a coacervate-vesicle transition. This resulted in the morphological transformation of the proteinosome-in-coacervate system to a vesicle-in-proteinosome organization. In other studies, autocrine signaling has been used to alter the shape or structure of artificial cells. Rossi and co-workers designed an artificial cell model from mixed oleic acid/phospholipid giant unilamellar vesicle (GUV) that used autocrine signaling for self-division, via urease-mediated changes in pH coupled to application of an osmotic pressure gradient (Miele et al., 2020). Huang et al. constructed a polymersome-based artificial cell that used autocrine signaling to provide structural rigidity to itself based on *in situ* alkaline phosphatase-driven production of Fmoc-TyrOH hydrogel network (Huang et al., 2014). This layer of hydrogel on the membrane also acted as a cell wall providing protection against unfavorable extracellular environments containing proteases.

### 4.2 Juxtracrine Signaling

Juxtracrine or contact-dependent signaling is a type of communication where the sender cell is physically in direct contact with the receiver cell, which implies that the typical response time scales are very short. In artificial cell research, this type of communication has mostly been explored using lipid vesicles and pore-forming proteins/peptides to regulate the exchange of molecules between adjacent vesicles and with the external environment (Figure 2B). The low permeability of the lipid bilayers to polar molecules allows channelling of molecule exchange via pore forming proteins/peptides enabling selective communication between adjacent vesicles to establish synthetic cell networks.

Bayley and co-workers pioneered the work of multisomes where networks of aqueous droplets are encapsulated within small oil droplets suspended in water and are capable of exchanging molecules with the external aqueous environment as well as among themselves (Villar et al., 2011). The aqueous droplets are coated with lipid monolayers joined through interface bilayers and the communication across the water droplets and with the external aqueous solution is mediated by α-hemolysin protein pores (Villar et al., 2013). Similarly, (Elani et al., 2013) showed a binary vesicle network constructed through the droplet interface bilayer (DIB) technique where α-hemolysin enabled free diffusion of signaling molecules. They went on further to construct multicompartment lipid bilayer vesicles (Elani et al., 2014; Elani et al., 2016; Bolognesi et al., 2018) where each compartment hosted a single step within a three-step enzyme cascade and α-hemolysin pore was used to allow exchange of signaling molecules/intermediates to establish communication within compartments and with the environment. In a recent study, Han and coworkers (Wang et al., 2019) constructed a hemifused pair of sender and receiver giant unilamellar lipid vesicles (GUV) encapsulating GOx and HRP, respectively, and used a pore forming peptide, melittin, to transport glucose into the sender cell where it was oxidized by GOx to produce hydrogen peroxide that diffused across the lipid membrane into the receiver cell containing HRP as a signal. In comparison, Oscar Ces and coworkers (Bolognesi et al., 2018) constructed vesicle networks where the diffusion of signaling molecules occurred across two adhering bilayers. Each bilayer was bridged by α-hemolysin protein pore to enable signaling across the two bilayers and selectively blocking the α-hemolysin pore molecule exposed to extracellular solution by heptakis (2,3,6-tri-O-methyl)-β-cyclodextrin (TRIMEB).

Other than protein pore-mediated direct signaling, there are few examples where proteinosomes have been cross-linked using covalent bonds to form synthetic tissues exhibiting internal signaling (Figure 2B). Gobbo et al. used covalent biorthogonal chemistry to crosslink proteinosomes to construct artificial prototissues (Gobbo et al., 2018) and recently showed direct signal exchange between covalently crosslinked GOx and HRP-loaded artificial cells to produce resorufin as final product (Galanti et al., 2021). In this case, the high molecular cut-off of the proteinosome membrane allows fast internal signaling within the synthetic tissues.

### 4.3 Paracrine Signaling

Paracrine signaling is the mode of communication where sender and receiver cells are in proximity (L≥d) . The timescales of signaling are typically 100-fold longer than autocrine and juxtacrine for a given diffusible molecule due to the ca. 10-fold longer distances across which the signal needs to diffuse and build up to a threshold concentration to elicit a response from the receiver cell. The size of the signaling molecule is also crucial as it determines the rate of diffusion which impacts timescales involved as well. For example, typical diffusion coefficients for molecules (r = 0.1 nm), proteins (r = 1 nm) and DNA strands (r = 10 nm) are D ∼ 2 × 10^3^, 2 × 10^2^ and 2 × 10^1^ μm^2^ s^−1^, respectively, so that in 1 s molecules, proteins and DNA will have diffused ca. 50, 15, and 5 μm away from the sender cell, respectively. We limit our discussion to paracrine signaling between artificial cells; examples where artificial cells communicate with living cells have also been reported (Toparlak et al., 2020) and have been reviewed elsewhere (Chen C. et al., 2021).

Paracrine signaling has been mostly demonstrated by using linear two step enzyme cascades where each step involved in the cascade is hosted within different cells and the diffusion of reaction intermediates constitute signaling between cells (Figure 2C). Most of the studies have shown communication between two types of cells only with limited range of responses, which predominantly involved triggering chemical reactions generating a fluorescent product. Mason et al. utilized a GOx-HRP enzyme cascade to show paracrine signaling where sender and receiver cells were copolymer-stabilised coacervate droplets (Mason et al., 2017). Similarly, Sun et al. demonstrated communication between colloidosome protocells where sender colloidosomes containing GOx generate hydrogen peroxide (H_2_O_2_) as a signal that is received by another colloidosome to assist in the formation of a polymer membrane (Sun et al., 2016). Here, signaling impacted morphological changes to the receiver cell, which enabled temperature-based regulation of transport across its membrane. Estirado et al. designed a DNA-decorated nanoscaffold immobilised within a coacervate core that was stabilized by a polymeric membrane to demonstrate DNA-strand displacement-based signaling (Magdalena Estirado et al., 2020).

Other than enzyme cascades and DNA-strand displacement reactions, some studies have also used transcription-translation (TX-TL) genetic circuits as the biological machinery for driving signalling. For example, Tang et al. described the design and assembly of a gene-mediated chemical communication pathway between lipid vesicle transmitters and proteinosome receivers (Tang et al., 2018). In another study, Adamala et al. demonstrated paracrine-like communication between two lipid vesicles used as minimal synthetic cell which they termed as synells (Adamala et al., 2017). The sender synells secreted doxycycline (DOX) and isopropyl-β-D-thiogalactoside (IPTG) as signaling molecules upon activation of encapsulated TX-TL genetic circuits to produce α-hemolysin. The receiver synells also contained TX-TL genetic circuits which were triggered by the signaling molecules to express firefly luciferase. In another noticeable study, Devaraj and coworkers showed communication between polymeric microcapsules containing a clay-DNA hydrogel core where proteins were used as signaling molecules (Niederholtmeyer et al., 2018). The membranes were permeable to TX-TL machinery supplied in the external medium, which allowed free exchange of mRNA and proteins synthesised using DNA of each cell to enable signaling via protein molecules.

During paracrine signaling, signal dilution may limit the distance over which communication takes place. Recent studies have been geared towards signal amplification to circumvent such limitation (Figure 2C). Buddingh et al. showed paracrine signaling over longer distances by allosteric amplification within lipid vesicle-based artificial cells, which facilitated the propagation of signaling fronts in communities of sender and receiver cells (Buddingh’ et al., 2020). Such amplification of signaling was also shown by Tom de Greef et al. where they employed DNA-strand displacement reactions to implement signaling between DNA-strands immobilized within proteinosome sender and receiver cells (Joesaar et al., 2019). The complex topology of reactions enabled by DNA-strand displacement technology allowed them to demonstrate 3-step cascade reactions, signal amplification, bidirectional negative feedback loop and logic gate operations. Bi-directional feedback loops are common features of developmental signaling in real cells (Freeman, 2000) and Joesaar et al. have been able to implement them to enhance the functionality of artificial cells.

Other than the size of the signaling molecule, there are other factors that influence the length scales involved in paracrine signaling such as receiver cell density, consumption rate of signaling molecule by receiver cells, signal degradation and permeability of receiver cell to signaling molecule. Tom de Greef and coworkers have looked into the details of the effects of all the above parameters on paracrine signaling by using DNA-strand displacement reactions which can be triggered by light, microfluidic traps to control receiver cell density, concentration of immobilized DNA strands within proteinosomes to control signal consumption per receiver cell and the presence of exonuclease to control signal degradation (Yang et al., 2020).

### 4.4 Endocrine Signaling

Endocrine signaling is another distance-based communication between cells where sender and receiver cells are very distant from one another (L≫d) and the signaling molecules find their way to the receiver cells via simple diffusion coupled with flow of the external medium. This is generally observed in multicellular organisms with circulatory systems, where the endocrine signaling factors (hormones) are secreted into the blood stream and transported to various target organs. In artificial cell systems, there are only a few studies where flow has been employed to carry signaling molecules from the sender to the receiver cells. Liu at al. demonstrated communication under unidirectional flow between coacervate-based colonies immobilized in agarose hydrogels (Liu et al., 2020). The sender cell colony was constituted using coacervates containing TiO_2_/Ag nanoparticles that can produce Hydrogen peroxide (H_2_O_2_) as a signaling molecule by photocatalytic reduction of dioxygen. The signaling molecule was transported unidirectionally from sender to receiver colony with the help of a subtle flow induced by placing a filter paper at one end of the hydrogel colony and adding water slowly at the opposite end. In the receiver cell colony, coacervates were sequestered with G4-hemin DNAzyme that processed the signaling molecule to produce the red fluorescent product resorufin by oxidation of Amplex red. Further oxidation of resorufin to resazurin which is non fluorescent allowed the demonstration of signaling front in the form of a travelling fluorescence band.

The development of microfluidics tools will open new perspectives to study endocrine signaling in articifical cells. Microfluidics has indeed already offered ways to study living cell-cell signaling and biochemical cross-talk between cells by hosting them in spatially defined regions, separating them using different types of porous barriers and connecting different colonies via flow in microfluidic channels. These studies have garnered a lot of attention leading to rapid development of Organ-on-a-Chip (OoC) and human-on-a-chip devices for toxicity screening, drug metabolism, pharmacokinetics, cancer metastasis and ADMET profiling (ADMET: Absorption/Distribution/Metabolism/Excretion/Toxicity) (Park et al., 2018; Picollet-D’hahan et al., 2021). With the rapid strides of artificial cell research, such microfluidic technologies will become more relevant to the system level design of consortia of prototissues or protoorgans that are completely built out of synthetic or hybrid materials and capable of complex coordinated functions and behaviours.

## 5 Dynamic Control Over Communication in Artificial Cells

Chemical signaling in living cells is adaptive. The design of stimuli-responsive assemblies and the development of new tools to program and manipulate artificial cells provides a first step towards dynamic signaling regulation in synthetic micro-compartments. In this section, we exemplify such emerging strategies by classifying them depending on which element of the communication chain (sender, signaling distance or receiver) they act on.

### 5.1 Switchable Sender Activation

**FIGURE 3:**
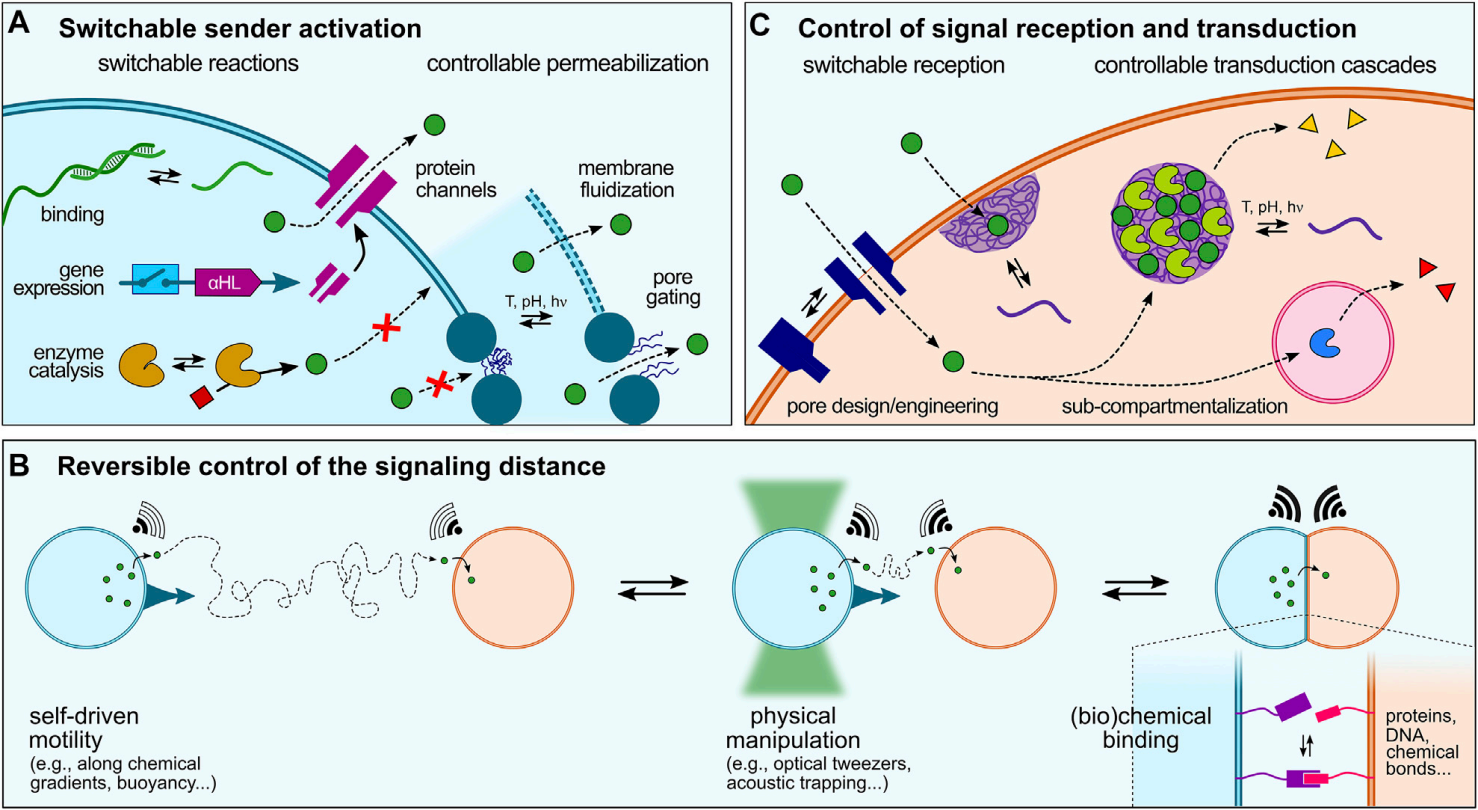
Dynamic control over artificial cell signaling. **(A)** Various strategies can be implemented to regulate signal production or release that can be schematically split in two categories: switchable reactions, such as sequence-specific ssDNA binding and release, switchable gene expression (e.g., using riboswitches), or activation of enzyme reactions, and controllable permeabilization, via gene-directed *in situ* synthesis of protein pores, membrane fluidization and formation of defects in continuous lipid- or polymer-based membranes, or pore gating in discontinuous membranes. **(B)** Reversible modulation of artificial cell-cell communication could also be achieved by dynamically changing the distance between sender and receiver cell, e.g. using physical manipulation or chemical binding strategies, or by exploiting the motility of artificial cells. **(C)** Signal reception and transduction within receiver cells can last be regulated by switchable reception, e.g., using stimuli-responsive pores or strategies to enhanced signal transduction across membranes such as membrane-addressed coacervates, and programmable transduction pathways, e.g. using specific addressing into sub-compartments.

In living cells, molecular signals are generated by *in situ* biochemical reactions, which either diffuse out of the cell passively (e.g., through the semi-permeable lipid membrane or pore channels) or are excreted via specific processes (e.g., exocytosis) precisely synchronized in space and time. In artificial cells, remote control over signal production and release can be achieved using stimuli-responsive reactions and assemblies (Figure 3A).

Activation of biochemical reactions in synthetic cells typically starts by the addition of specific molecules, such as enzyme substrates or gene inducers that can be viewed as primary triggers. Rationally designed molecules have thus been developed to trigger the activation of biochemical reactions in synthetic cells. Recent examples include the use of small molecules that activate gene-directed production of proteins via synthetic riboswitches (Lentini et al., 2014; Dwidar et al., 2019). A greater temporal control over signal production or release can be achieved using physical stimuli. In a seminal study, de Greef and collaborators demonstrated light-controlled release of single-stranded DNA signals from a sender proteinosome upon photocleavage of longer strands into shorter ones and their resulting dissociation from a complementary ssDNA anchor (Yang et al., 2020).

Sender activation can also be achieved upon permeabilization to trigger the release of encapsulated signaling molecules. In the case of continuous membranes (liposomes and polymersomes), this has been recently shown by Haylock et al., who demonstrated regulation of communication between ternary network of droplet interface bilayers via controlled activation of protein pores using a chemical activator (Haylock et al., 2020). *In situ* gene-directed expression of pore proteins has also been exploited to trigger signaling (Findlay et al., 2016). Bayley and coworkers engineered a tissue mimic by 3D-printing PURE-based TX-TL-containing droplets that produced α-hemolysin pore proteins upon light-activation, which were then spontaneously incorporated into specific bilayer interfaces to mediate rapid, directional electrical communication between subsets of artificial cells, reminiscent of neural transmission in living cell (Booth et al., 2016). Tang et al. showed that intravesicular α-hemolysin expression initiated by the addition of a transcription inducer within TX-TL-containing liposomes triggered membrane pore formation, which allowed the release of encapsulated glucose and the activation of a GOx-HRP cascade enzymatic reaction in receiver proteinosomes (Tang et al., 2018). Using a similar approach, Adamala et al. created controlled communication pathways between populations of synthetic cells based on two component circuits built by mixing two populations of sensors and reporters liposomes. The sensor liposomes contained IPTG (isopropyl-β-D-thiogalactoside) and the arabinose inducible gene for α-hemolysin. These liposomes thus sensed arabinose and released IPTG by expressing α-hemolysin channels (Adamala et al., 2017). Going a step closer to living systems, a recent study has demonstrated temperature-induced exocytosis of polymersomes in lipid-based vesicles multi-compartments based on thermally induced changes in polymer amphiphilicity (Chen H. et al., 2021).

Release of molecules through lipid or polymer membranes has also been realized by perturbing the membrane organization in response to environmental stimuli. In an example, Lecommandoux and colleagues spatially separated incompatible enzymes involved in a cascade reaction into distinct polymer-based compartments, and temperature was used to selectively activate one cascade over the other by membrane fluidization (Peters et al., 2014). Ces and co-workers used light to trigger the formation of pores in lipid vesicles via diacetylene polymerization, which allowed the release of a molecular substrate and subsequent activation of an enzyme reaction (Hindley et al., 2018). In comparison, the permeability of discontinuous membranes can be tuned by stimuli-responsive pore gating. Mann et al. demonstrated this behaviour in colloidosomes via crosslinking and covalent grafting of a pH-responsive copolymer to generate an ultrathin elastic membrane that exhibited selective permeability to small molecules depending on pH-mediated changes in the charge of the copolymer coronal layer (Li et al., 2013).

Control over molecular exchange in membrane-free droplets often proves more complex due to the absence of a barrier (Mitrea et al., 2018; Alberti et al., 2019). Studies inspired by the selective sequestration of guest biomolecules in phase-separated biomolecular condensates found in living cells are now exploring the use of specific binding motifs to selectively sequester and release biomolecules in such droplets in response to external cues. van Hest et al. reported a coacervate-based protocellular platform that utilizes the well-known binding motif between Ni^2+^-nitrilotriacetic acid and His-tagged proteins to exercise a high level of control over the loading of biologically relevant macromolecules (Altenburg et al., 2020). Sequence-dependent light-switchable DNA recognition has also been used very recently to demonstrate light-driven release and uptake of azobenzene-functionalized DNA strands in coacervate microdroplets (Zhao et al., 2022).

### 5.2 Reversible Control of the Signaling Distance

We have discussed in Section 4 different modes of signaling depending on the spatial distribution of sender and receiver synthetic cells. In all these examples, the distance between synthetic cells was fixed throughout the studies. In comparison, living cells can move and dynamically adapt their spatial organization or number density, which affects the inter-cell distance and therefore the ability of cells to communicate with each other. To the best of our knowledge, there has not yet been a clear attempt to dynamically change the distance between sender and receiver artificial cells and demonstrate how this would affect chemical signaling. However, several existing tools to manipulate or program artificial cells could be used to modulate the distance between two communicating artificial cells (Figure 3B), which we briefly discuss below.

Physical manipulation of artificial cells has been demonstrated using magnetic fields, acoustic patterning or optical tweezers. Li et al. magnetically assembled GUVs into various microstructures with spatially encoded configurations using a stainless steel mesh with patterned microwells in a paramagnetic solution media, which was harnessed for engineering cascade enzyme reactions (Li et al., 2020). Mann et al. used micro-arrays of acoustically trapped GUVs for the spatial positioning and signaling of enzyme reactions. By trapping colonies of heterogeneous GUV populations containing either GOx or HRP, they produced spatially distributed communities of artificial cells capable of localized enzyme-mediated chemical signaling triggered by a pore-forming peptide (melittin) inserted into the lipid membrane (Wang et al., 2019). Optical tweezers have been used by Bolognesi et al. to controllably bring together vesicles in a targeted manner. Patterning membranes with proteins and nanoparticles facilitated material exchange between compartments and enabled laser-triggered vesicle merging, permitting protein expression by delivering biomolecular reaction components (Bolognesi et al., 2018). These approaches could be used to change the spatial positioning of artificial cells and in turn regulate their communication ability.

Non-covalent chemical interactions could also be harnessed to dynamically control the distance between communicating artificial cells. For instance, Palivan et al. showed how clusterization of enzyme-containing nanometer-sized polymersomes tethered together by hybridization of complementary single-stranded DNA (ssDNA) exposed on their surface could be harnessed to control a cascade enzyme reaction. The distance between the catalytic nanocompartments within a cluster could be easily controlled by modifying the length of DNA strands which affects the overall performance of the cascade reaction (Liu et al., 2016). Such a strategy could be adapted to larger, cell-sized compartments and reversibility provided by addition of free DNA strands to favor detachment of the compartments. Stimuli-responsive interactions offer another promising approach to reversibly control the distance between compartments. Notably, the Wegner group demonstrated the use of light-responsive protein interactions to achieve photo-controllable binding of GUVs, which resulted in the activation of signaling between two artificial cells due to the shorter distance between them upon binding (Chakraborty and Wegner, 2021).

Moving towards more autonomous systems, artificial cell motility could also be exploited to control population density and therefore tune the distance between sender and receiver cells. Examples of artificial cells capable of motility include catalase-containing organoclay/DNA semipermeable microcapsule, which in the presence of hydrogen peroxide exhibits enzyme-powered oxygen gas bubble-dependent buoyancy (Kumar et al., 2018); layer-by-layer (LbL) capsosomes functionalized with an asymmetric layer of Pt nanoparticles (PtNPs), which are capable of free motion thanks to the catalytic production of oxygen bubbles in the presence of hydrogen peroxide; or asymmetrical polystyrene-block-poly (ethylene glycol) (PS-b-PEG) polymersomes encapsulating various catalysts and capable of nanoscale motion in the presence of appropriate fuel (Wilson et al., 2012a; b).

### 5.3 Control of Signal Reception and Transduction

To achieve a desired response and ensure proper signal transduction, the signal needs to be interpreted by the receiver. Living cells rely on complex transduction cascades to decode molecular signals binding to receptors, and ultimately produce a precise response at a specific location, such as expression of a given gene in the nucleus. Signal transduction also often includes an amplification step to relay low concentrations of secreted chemical signals into an effective intracellular process (Rosenbaum et al., 2009). Such complex processes have not yet been matched in artificial cells (Figure 3C). In most (if not all) current examples, signaling molecules reach receiver cells and activate a single–often simple–reaction (e.g. to produce a fluorescent reporter), but is not specifically addressed to a spatial location, nor amplified. As discussed above, allosteric amplification of a signal in a population of artificial cells has been recently demonstrated by van Hest et al. (Buddingh’ et al., 2020), paving the way to a finer control over receiver response.

Regarding specific addressing of signals, sub-compartmentalization is used by cells for sustaining the multiplexed circuitry enabling complex signal processing and generation of integrated responses. Cells can shift metabolic flux in response to diverse signals by redistributing existing enzymes into organelles without affecting total enzyme quantities. The idea of engineering synthetic organelles to co-localize the components of an engineered metabolism has gained considerable interest in recent years. Reproducing such complex pathways in artificial cells can be achieved by building multi-compartmentalized ensembles (Lee et al., 2018; Belluati et al., 2020; Ip et al., 2021; Wen et al., 2021). In a recent example, Robinson et al. developed a microfluidic platform to produce monodisperse multivesicular vesicles (MVVs) to serve as synthetic mimics of eukaryotic cells. The MVVs contained three separate inner compartments encapsulating a different enzyme, and directed chemical communication between the compartments was achieved via the reconstitution of size-selective membrane pores (Shetty et al., 2021).

Membraneless organelles have been shown to be equally important to well-studied membrane-bounded organelles for cellular dynamics, regulation and the generation of high-fidelity cellular responses. An exciting example of membraneless organelle engineering in living cells was recently provided by the group of Toettcher who used optogenetic tools to control the reversible formation of condensates in cells and achieve *in vivo* metabolic control (Zhao et al., 2019). This type of strategy could be generalized to artificial cells. Coacervate microdroplets produced by associative liquid-liquid phase separation between oppositely charged polyions are used to mimic functions of cellular biomolecular condensates. These coacervates can now be engineered to be formed and dissolved in response to external cues, such as pH or temperature changes, or even light (Deng and Huck, 2017; Martin, 2019). For instance, Dekker et al. emulated the 2D organization of internal condensates on membranes (Deshpande et al., 2019). Using an on-chip microfluidic method, they could control and study the formation of membraneless organelles within liposomes via a transmembrane proton flux induced by a stepwise change in the external pH (Last et al., 2020). Using photo-switchable azobenzene, several groups designed photo-responsive coacervate micro-droplets that can form in the dark and disassemble or reassemble under UV and blue light irradiation respectively due to the *cis* to *trans* azobenzene photoisomerization (Martin et al., 2019; Huang et al., 2021; Lafon and Martin, 2021). These coacervates were recently integrated into semi permeable proteinosomes to build hierarchical protocells that could sense and respond to extracellular signals permitting to design Boolean logic gates (Mu et al.).

## 6 Emergent and Collective Behaviours Enabled by Signaling

There is an increasing number of examples where collective behaviours using signaling circuits are becoming more complex. A few emerging examples are highlighted below.

### 6.1 Quorum Sensing

**FIGURE 4:**
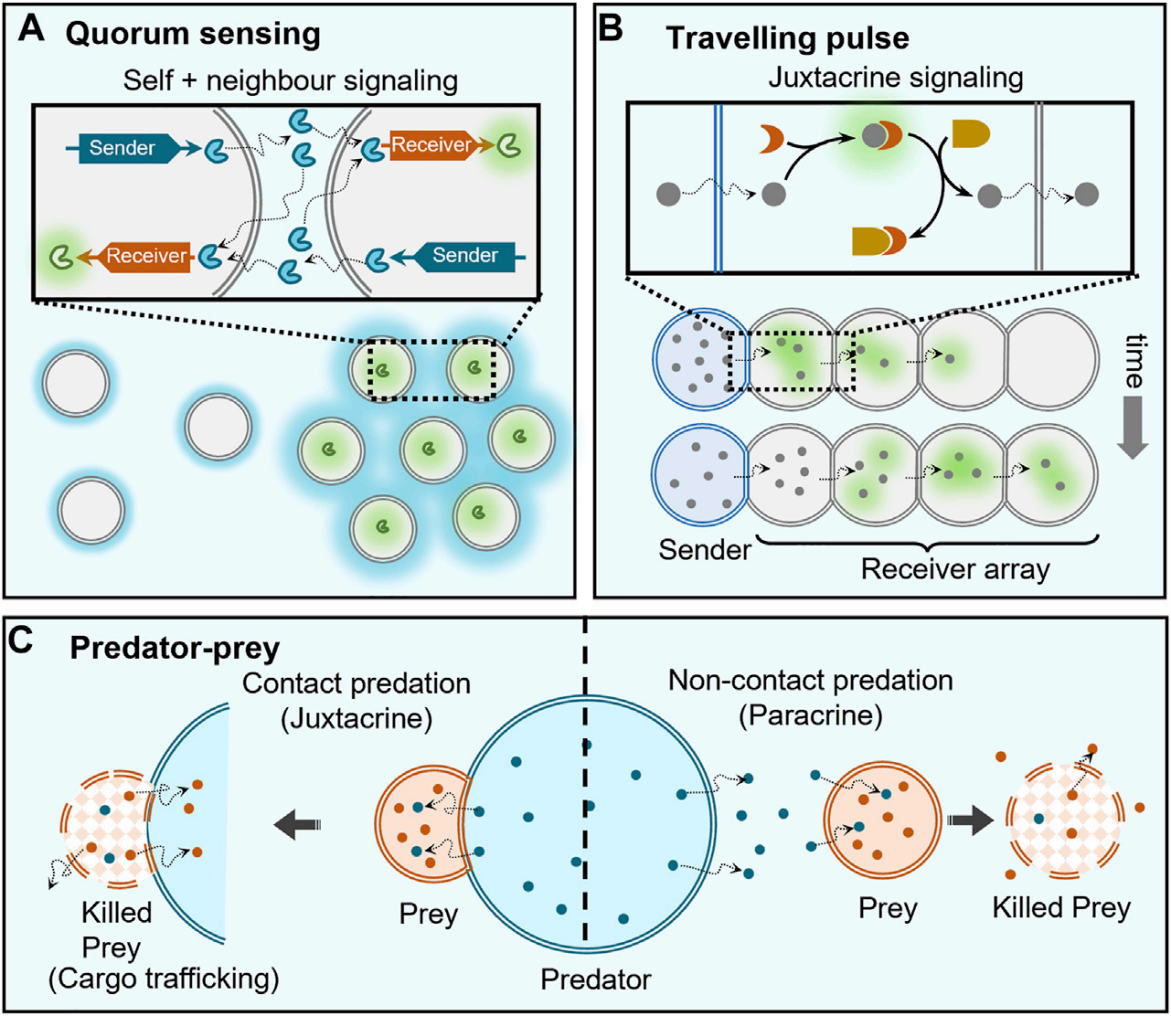
Collective behaviours enabled by cell signaling. **(A)** Artificial cells capable of population density-dependent behavior, i.e. quorum sensing, contain both sender and receiver DNA templates so that they can both send and receive protein signals (blue) to trigger the expression of a green fluorescent protein at high population densities, where the loss of self-generated signaling molecules by dilution is countered by the receipt of signals secreted by neighbouring cells (indicated by strong blue hues around clustered cells). **(B)** Travelling pulse behaviour in artificial cells can be realized by propagation of signals from a single sender cell through an array of receiver cells, where the signaling molecules are capable of diffusing across the membranes between the cells and are coupled to a feedforward genelet circuit. **(C)** Two types of predator-prey behaviour are observed depending on the distance between predator and prey: contact predation, when they are in direct contact allowing immediate degradation (killing) and trafficking of contents of prey cell, and non-contact predation, when they are separated by small distances ( L≥d ) leading to slow degradation of prey cell.

One of the emergent properties of signaling is quorum sensing where behaviour is regulated based on the fluctuations in cell-population density (Figure 4A). Here, the cells are self-signaling (autocrine) as well as signaling to like-cells in the immediate vicinity (paracrine). This collective behaviour is extensively present in mammalian and bacterial cells (Ng and Bassler, 2009), and also observed in organisms of larger length scales like ants that are known to use quorum sensing for eliminating multiple nests (Grivennikov and Karin, 2008). Pioneering studies by Mansy and Stano have extended such a quorum sensing behaviour to populations consisting of both artificial and living cells (Lentini et al., 2017; Rampioni et al., 2018; Toparlak et al., 2020). In populations consisting of solely artificial cells, to the best of our knowledge, Niederholtmeyer et al. were the first to report artificial quorum sensing using polymeric compartments with a clay/DNA hydrogel core that used a transcription-translation (TX-TL) machinery present in the external medium to synthesise different proteins based on the DNA in the hydrogel core (Niederholtmeyer et al., 2018). The latter contained two DNA templates, a sender template for the production of T3 RNA polymerase (T3-RNAP) and a receiver template capable of T3-RNAP-dependent expression of a green fluorescent protein. As the cells also contained both sender and receiver circuits, they could both send and receive signals themselves, which allowed them to achieve density dependent expression of green fluorescent proteins. The reason for such a behaviour is dilution of the activator T3-RNAP protein at low artificial cell density that reached the critical threshold when a sufficient population density was achieved. Mimicking of quorum sensing based on signaling in artificial cells will allow construction of more life-like materials in the future (Leslie, 2018).

### 6.2 Pulses and Synchronization

The complexity enabled by signaling can be increased by employing genetic circuits to access different reaction topologies. In a noticeable work, Dupin et al. used chemical signals capable of activating genetic circuits within water-in-oil droplet-based multi-compartments to demonstrate communicating networks of artificial cells capable of generation of travelling pulses as well as differentiation (Dupin and Simmel, 2019). Here, α-hemolysin protein pores were used to selectively transport signaling molecules between a central sender cell and neighbouring receiver cells, trigger *in vitro* gene circuits allowing for positive feedback and demonstrate stochastic differentiation of receiver cells. Additionally, they implemented an incoherent type-1 feed-forward loop genelet circuit to demonstrate propagation of a pulse from a single sender cell to a 1D/2D array of receiver cells. Sender cell released a dormant fluorophore 3,5-difluoro-4-hydroxybenzylideneimidazolineone (DFHBI) that was captured by RNA present in receiver droplets to trigger fluorescence, but this also activated a *ds*DNA template which displaced DFHBI to cause the fluorescence to decay. This activation and deactivation of fluorophore propagated a fluorescent pulse through the array of receiver droplets (Figure 4B).

### 6.3 Predatory-Prey and Killer Cell Behaviour

Paracrine signaling has been used to induce deterioration of the receiver cell, and therefore demonstrate predator-prey or killer cell behaviours (Figure 4C). Raghavan et al. demonstrated killer cell behaviour where the killer capsule containing GOx secreted gluconate to degrade neighbouring alginate beads (targets) (Arya et al., 2016). Qiao et al. showed predatory-prey behaviour between two different communities of artificial cells (Qiao et al., 2017). This ambush predation was mediated through electrostatic interactions between the “predator” coacervate cell and proteinosome “prey” cells. The coacervate predator cells contained proteases capable of degrading the proteinosome membrane to capture their contents and imbibe their properties. The same group constructed a response-retaliation behaviour in a hybrid artificial cell community (Qiao et al., 2019).

Thanks to a better control over chemical and physical interactions between artificial cells; we anticipate that other types of collective behaviours will be developed in the years to come, including cooperativity and symbiosis or parasitism.

## 7 Future Challenges

### 7.1 Quantification: Towards an Information Theory of Molecular Communication

As exemplified above, the vast majority of studies on artificial cell communication are still highly phenomenological and often lack quantitative insight. There is therefore a need for more quantitative studies to test hypotheses on molecular communication and ultimately develop a comprehensive theoretical framework that accounts for the discrete diffusion of molecules, molecular background noise, molecule degradation etc.

Notably, compared to conventional means of communication based on electromagnetic signals, chemical signaling has low energy requirements and exhibit high specificity. Hence, information can be readily encoded in the chemical nature of the molecules themselves, and does not necessarily requires complex encoding processes (e.g., frequency or intensity modulation). Chemical signaling is yet usually short range (except when flows of the surrounding medium are involved), slow, and stochastic due to the possible degradation of molecules, their diffusion, and the presence of a high background molecular noise (Okaie and Nakano, 2020; Magarini and Stano, 2021). These peculiarities may explain why, despite an in-depth phenomenological knowledge on biological chemical signaling, a rigorous information and communication theory of chemical communication is still lacking. Pioneering efforts initiated in the early 2000s have led to the emergence of the field of “molecular communication” to adapt the well-established communication theories used for telecommunication to chemical communication by combining biology and computer networks (Caterina et al., 1997; Okaie and Nakano, 2020). While new mathematical tools are needed to take into account the complexity of molecular communication and describe with a better accuracy the discrete diffusion of molecules, the general Shannon theory for telecommunication has been successfully adapted to chemical signals (Katzmann et al., 2002; de Luis et al., 2021).

Further systematic studies, e.g., of the impact of sender-receiver geometry on signaling, should be performed. Precise or targeted manipulation of artificial cells using magnetic field, optical tweezers, ultrasound waves, micromanipulators or 3D printers are promising tools for such a purpose. The design of new types of compartments with variable membrane permeability is also an interesting perspective to gain deeper insight into molecular communication.

### 7.2 Complexity, Programmability, Multiplexing

Although most of the studies on artificial cell communication have been based on linear or cascade modes of communication, we have seen above that recent works try to increase the complexity of the signaling pathway by programming feedback, e.g., based on DNA strand displacement and genetic circuits. Future steps would be achieving orthogonal signaling, namely processing multiple signals at the same time without cross-talk, combining different types of signaling (e.g., autocrine and paracrine), or understanding how competition for the signal among homogenous or heterogenous colloidal communities affects signal transmission. Recently, Bayley and coworkers took a step in this direction by developing a three-compartment multisome system stabilised by lipids in aqueous environment that has ability to receive and process signals in parallel (Cazimoglu et al., 2021). This parallel signaling was achieved by using magnesium (Mg^2+^) and lactose as input signal that produced a fluorescence output in endonuclease EcoRI containing droplet and glucose production β-galactosidase containing water droplet, respectively. We anticipate that future studies will be geared towards developing more intricate signaling networks among consortia of artificial cells by a finer programmability and remote control over signal production, transmission or reception.

Complexification of signaling responses may also come with complexification of the compartments themselves, e.g., via the design of dynamical systems capable of reconfiguration, restructuration, division, growth, differentiation (Gaut et al., 2022), etc., but also by complexification of the reactions used for signaling, e.g. coupling diverse reactions (gene expression, enzyme activity, DNA strand displacement, etc.), and using external stimuli to precisely control each of them.

## 8 Conclusion

In this review, we have illustrated how this rapidly emerging field of communication in artificial cells has progressed in the last decade with a majority of the strides being taken in the last 5 years alone. It started with more conventional self-modification of behaviour (autocrine signaling), progressed to paracrine/juxtacrine signaling and went further to achieve dynamic adaptive signaling at a population level to enable complex collective behaviours such as quorum sensing, travelling waves and pulses and differentiation of artificial cells. Different modes of communication were also explored such as linear, cascade, bidirectional and networked communication. Nevertheless, artificial cell signaling is still in its infancy when compared to its counterpart in living cells where complex signaling networks operate to control and coordinate behaviour at different length scales enabling organization, maintenance and multiplication of multicellular organisms or architectures. The importance of signaling in living systems is representative of its role in the development of artificial cell systems, to make them more autonomous, exhibit complex spatiotemporal patterning, differentiation and functional regulation. It could lead to the development of a new generation of smart colloidal agents, such as theranostic agents capable of smart multi-cycle delivery of payloads upon receiving appropriate biological cues. Though we have not reviewed the research regarding artificial cell communicating with living cells in this review, it also presents an important facet for artificial cell technologies. We can also envision a future where living cells are cultured in the presence of artificial cells to fabricate hybrid implants for regenerative medicine where the integrated artificial cells can perform functions of support, maintenance and regulation of the living cells in the implant and in the vicinity.
